# Transcriptional Profiling of Peripheral Blood Mononuclear Cells in Pancreatic Cancer Patients Identifies Novel Genes with Potential Diagnostic Utility

**DOI:** 10.1371/journal.pone.0017014

**Published:** 2011-02-10

**Authors:** Michael J. Baine, Subhankar Chakraborty, Lynette M. Smith, Kavita Mallya, Aaron R. Sasson, Randall E. Brand, Surinder K. Batra

**Affiliations:** 1 Eppley Institute for Research in Cancer and Allied Diseases, University of Nebraska Medical Center Omaha, Omaha, Nebraska, United States of America; 2 Department of Biochemistry and Molecular Biology, University of Nebraska Medical Center, Omaha, Nebraska, United States of America; 3 Department of Biostatistics, University of Nebraska Medical Center, Omaha, Nebraska, United States of America; 4 Department of Surgery, University of Nebraska Medical Center, Omaha, Nebraska, United States of America; 5 Division of Gastroenterology, University of Pittsburgh School of Medicine, Pittsburgh, Pennsylvania, United States of America; Institut Pasteur, France

## Abstract

**Background:**

It is well known that many malignancies, including pancreatic cancer (PC), possess the ability to evade the immune system by indirectly downregulating the mononuclear cell machinery necessary to launch an effective immune response. This knowledge, in conjunction with the fact that the trancriptome of peripheral blood mononuclear cells has been shown to be altered in the context of many diseases, including renal cell carcinoma, lead us to study if any such alteration in gene expression exists in PC as it may have diagnostic utility.

**Methods and Findings:**

PBMC samples from 26 PC patients and 33 matched healthy controls were analyzed by whole genome cDNA microarray. Three hundred eighty-three genes were found to be significantly different between PC and healthy controls, with 65 having at least a 1.5 fold change in expression. Pathway analysis revealed that many of these genes fell into pathways responsible for hematopoietic differentiation, cytokine signaling, and natural killer (NK) cell and CD8+ T-cell cytotoxic response. Unsupervised hierarchical clustering analysis identified an eight-gene predictor set, consisting of *SSBP2, Ube2b-rs1, CA5B, F5, TBC1D8, ANXA3, ARG1,* and *ADAMTS20,* that could distinguish PC patients from healthy controls with an accuracy of 79% in a blinded subset of samples from treatment naïve patients, giving a sensitivity of 83% and a specificity of 75%.

**Conclusions:**

In summary, we report the first in-depth comparison of global gene expression profiles of PBMCs between PC patients and healthy controls. We have also identified a gene predictor set that can potentially be developed further for use in diagnostic algorithms in PC. Future directions of this research should include analysis of PBMC expression profiles in patients with chronic pancreatitis as well as increasing the number of early-stage patients to assess the utility of PBMCs in the early diagnosis of PC.

## Introduction

Pancreatic cancer (PC) remains a lethal malignancy with an overall five-year survival rate of only about 5% [1]. A significant contributor to the poor prognosis of PC patients is the failure to detect the tumor at an early and potentially resectable stage. It is estimated that only 8% of PC cases are diagnosed with tumors localized to the pancreas, while only 15–20% are considered resectable. Further, of those patients who have had their tumor resected, only 20% live more than 5 years post-diagnosis [2]. The most common cause of death post-resection is distant metastases; local recurrence is rare. Although studies showing prolonged survival in PC patients are rare, it is unquestionable that early detection and resection of PC, especially in a localized state, would likely yield a significant increase in survival.

Designing an early diagnostic test for PC however, presents a particular challenge owing to the relative rarity of the disease and the fact that the disease often remains asymptomatic until an advanced stage. Ideally, an early diagnostic test for PC would be minimally invasive, and relatively inexpensive, while being sufficiently sensitive to identify all or most cases of PC. When combined with a highly specific confirmatory test, it could potentially permit the early identification of patients with resectable disease.

CA19-9 is currently the only marker approved by the FDA for use in PC. However, while CA19-9 is useful as a marker of disease burden, it lacks both sensitivity and specificity (approximately 80% and 73% respectively) as a diagnostic marker [3]–[8]. Nonetheless, it remains the gold standard against which every potential biomarker is compared. In recent years, several new promising biomarkers have emerged which can potentially detect early stage PC either in the tissues (MUC4, MUC1, CECAM1) or in blood (MIC-1, NGAL, telomerase and microRNAs) [9]. However, none of these potential biomarkers are free of significant imperfections, showing sensitivities and/or specificities that are either poor or inconsistent between studies. Thus, there is a clinical need for novel markers for the early diagnosis of PC.

Peripheral blood mononuclear cells (PBMCs) comprise the circulating mononuclear cells, including monocytes, T-cells, B-cells, and natural killer (NK) cells, and have emerged in recent years as surrogate markers of several diseases including inflammatory (e.g. preeclampsia, rheumatoid arthritis, and chronic pancreatitis) and malignant (chronic lymphocytic leukemia and renal cell carcinoma) diseases [10]–[14]. However, their role in the detection and prognostication of solid tumors remains limited. In the present study, we hypothesized that an alteration in the global gene expression profile of PBMCs occurs in patients with PC and identification of PC-specific gene subsets in PBMCs could be potentially useful in the early detection of this malignancy.

Recent developments have permitted the development of gene chips containing a set of disease specific genes for either the diagnosis or predicting prognosis of several malignancies including breast and esophageal cancers [15]–[18]. The results of our study suggest that an eight-gene predictor set (selected from 383 differentially expressed genes out of 39,200 genes) can distinguish between PC and healthy individuals with a sensitivity and specificity of 83% and 64% respectively.

## Materials and Methods

### Study population

The study of blood-based biomarkers in PC was approved by the Institutional Review Board (IRB) at the University of Nebraska Medical Center (UNMC) (IRB number 209-00). Written informed consent was obtained from all patients and controls before enrollment into the study. For this study, 26 PC patients and 33 age, race, and gender matched healthy controls were recruited. A total of 35 samples were obtained from the PC and 33 from the healthy group. Baseline demographic information for both groups is detailed in Table 1.

**Table 1:** Demographics of PC Patients and Controls used in the study.

*Characteristic*	*PC*	*Healthy Controls*
*Samples*		
Number of patients	26	33
Number of PBMC samples	35	33
*Gender*		
Male	13 (50%)	6 (18.2%)
Female	13 (50%)	22 (66.7%)
Unknown	0 (0%)	5 (15.2%)
*Age*		
Mean (±SD)	64.4 (±9.0)	55.7 (±6.1)
Unknown	0 (0%)	5 (15.2%)
*Race*		
Caucasian	21 (80.8%)	25 (75.8%)
Non-Caucasian	3 (11.5%)	3 (9.1%)
Unknown	2 (7.7%)	5 (15.2%)
*Stage at Diagnosis*		
Resectable (stage 1- 2a)-patients recruited into study	4 (15.4%)	N/A
Resectable (stage 1-2a)-blood samples drawn	6 (17.1%)	N/A
Non-resectable (stage 2b or higher)-patients recruited into study	20 (76.9%)	N/A
Non-resectable (stage 2b or higher)-blood samples drawn	27 (77.1%)	N/A
Stage unknown-patients recruited into study	2 (7.7%)	N/A
Stage unknown-blood samples drawn	2 (5.7%)	N/A
*Surgical Status*		
Pre-surgical	23 (65.7%)	N/A
Post-surgical	12 (34.3%)	N/A
*Chemotherapy Status*		
Pre-chemotherapy	15 (42.9%)	N/A
Post- Chemotherapy	20 (57.1%)	N/A
Undergoing Chemotherapy at time of Sample Collection	10 (28.6%)	N/A

Abbreviations: PC, pancreatic cancer; PBMC, peripheral blood mononuclear cells.

The diagnosis of PC was based on a positive biopsy of a pancreatic mass or a metastatic lesion. The PC patients were further classified as localized (stage 1 and 2a) or non-localized (stage 2b and higher), pre- or post-surgery, and pre- or post-chemotherapy. A patient was classified as being post-surgery if they had undergone a pancreaticoduodenectomy before the sample was drawn. All other samples, including samples from patients who never had surgery during the course of their disease, were classified as pre-surgery. Any sample drawn before the patient had undergone any chemotherapy for PC was classified as pre-chemotherapy. If the patient had ever had chemotherapy for PC, regardless of whether or not that patient was undergoing chemotherapy at the time the sample draw, the sample was classified as being post-chemotherapy. For patients in whom multiple samples were drawn on different dates, all samples were used in the data analysis unless explicitly stated in the results section.

PC staging was based on one of four criteria: 1) pathological staging post-surgery, 2) MRI/CT/ultrasound staging, 3) endoscopic staging, or 4) biopsy of metastatic disease.

### Isolation of total RNA from PBMCs

PBMCs were isolated from whole blood using the PharmLyse RBC lysis solution (BD, San Jose, CA) according to the manufacturer's instructions. Total RNA was extracted using the Qiagen RNAeasy RNA isolation kit (QIAGEN, Valencia, CA, USA) and then converted to cDNA using the SuperScript II cDNA synthesis kit (Invitrogen, Carlsbad, CA) according to a previously published protocol [19].

### cDNA microarray analysis of global gene expression profile of PBMCs

Microarray analysis was performed by the UNMC microarray core facility using established lab protocol on a Phalanx whole genome cDNA microarray containing 30,275 features probing for approximately 22,000 unique genes. A universal human reference (Stratagene, Cat: 740000, Cedar Creek, TX) was used as the reference against which all samples were normalized.

### Statistical Analysis

Log_2_ transformation was applied to all ratios followed by normalization to “center” each array using Lowess smoother through BRB ArrayTools developed by Dr. Richard Simon and Amy Peng [20]. Any gene in which the percent of spots missing or filtered out exceeded 50% was excluded. Duplicate spots were not averaged but treated as separate genes for analysis. Mixed effects models were then used to determine which genes were significantly differentially expressed between the PC and the healthy control groups, allowing for a 10% false discovery rate. Diagnosis group (cancer vs. normal) was included in the model as a fixed effect and a random subject effect was also included to account for multiple samples per person.

Hierarchical clustering analysis of the arrays based on similarity of expression profiles was performed using the normalized and log_2_-transformed data. Clustering was done using Gene Cluster version 3.0, using the “centered” Pearson correlation similarity metric and complete linkage clustering method, and visualized using Java TreeView.

### Validation of microarray data by Q-RT PCR

The microarray results were validated by quantitative real time PCR (Q-RT PCR). All Q-RT PCR reactions utilized SYBR green based chemistry. For validation, six of the most differentially expressed genes: 3 up-regulated (*ANKRD22, ANXA3, ARG1*) and 3 down-regulated (*FCER1A, GRAMD1C, and MS4A1*) by microarray were chosen. Validation was done in a randomly selected subset of the original samples (submitted for microarray analysis) that included nine healthy controls and twelve PC patients. The fold-change in gene expression was determined by the 2^-ΔΔCt^ method using the same human reference RNA as that employed in the microarray. To determine the correlation between the microarray and Q-RT PCR results, we calculated the median fold change in expression (for a given gene) for PC vs. healthy controls, and compared it to the fold change seen by microarray to determine whether the gene was still differentially expressed in the same direction.

### Correlation of gene signatures with clinicopathologic characteristics in PC

To determine if there is the differential expression of genes in PC patients correlates with patient characteristics, a mixed effects model was applied to the PC samples that were grouped according to the following criteria: surgical status (pre- vs. post-surgery), chemotherapy status (pre- vs. post-chemotherapy), history of type-II diabetes mellitus prior to the diagnosis of PC (present vs. absent), location of the tumor (head vs. body/tail), and stage of PC (localized vs. non-localized, and metastatic vs. non-metastatic). Significant genes were chosen based on an allowable false discovery rate of less than 10%. Stage 1a and 2a PC were considered localized, while stages 2b, 3, and 4 PCs were considered non-localized, and stage 4 tumors were considered metastatic. For two patients recruited into the study, information on tumor stage, tumor location, and history of type-II diabetes mellitus could not be obtained.

### Identification of a gene predictor set that distinguishes PC from healthy individuals

BRB-ArrayTools Version 3.8.0 was used to analyze all possible combinations of the 21,671 valid genes identified by microarray to determine whether a genetic signature could be identified that would distinguish PC patients from healthy controls with the optimum combination of sensitivity and specificity. The microarray data for 24 randomly chosen PC samples and 20 healthy controls was entered into the analysis. Genes to be selected for the predictor set were required to be significantly different between the PC and healthy control groups with a significance level of p≤0.0001 and with a fold difference expression between the two groups ≥1.5. Cross validation of the gene predictor set was repeated 1 times K-fold (K = 10). The gene predictor set arrived at through these methods was analyzed by various methods, including Compound Covariate Predictor, Diagonal Linear Discriminant Analysis, 1-Nearest Neighbor, 3-Nearest Neighbors, Nearest Centroid, Support Vector Machines, and Bayesian Compound Covariate Predictor. Of these, the Compound Covariate Predictor gave the best predictive capabilities using the gene predictor set and consequently used.

### Validation of the gene predictor set

Once the predictor set was established, it was validated in a second set of randomly selected PC and healthy samples. The statistician was blinded to the identity of the samples. Applying the cut-off obtained through the Compound Covariate Prediction method, the samples were classified as either “PC” or “non-PC”. The analyzer (M.B.) was then unblinded and the accuracy of the prediction determined by comparison with the actual diagnosis. We also applied the same equation to a subset of pre-chemotherapy pre-surgical PC patients to determine the ability of the predictor set to correctly classify patients into PC vs. non-PC. This is important as the influence of chemotherapy and/or surgery on the gene expression profile of PBMCs cannot be ruled out. Further, the latter group of patients represents the ideal patient population in whom the test, if validated would be applied in a clinical setting.

## Results

After normalization and filtering of the microarray data, 21,671 genes remained for analysis. Of these, 383 genes were found to have a significant differential expression between PC patients and healthy controls (Table S1). Of these, 65 genes were observed to have a differential expression ≥1.5-fold between the two groups (Tables 2–3).

**Table 2:** Genes Shown to be at Least 1.5 Fold Upregulated in PBMCs of PC Patients.

*Gene symbol*	*Unique id*	*FDR*	*Fold-change (PC/HC)*	*Gene Information and Normal Gene Function*
				
TMEM22	PH_hs_0038059	0.069	4.812	Transmembrane protein 22, Function unknown
MMP8	PH_hs_0024515	0.076	2.351	Neutrophil protein used to degrade type I, II and III collagens
ARG1	PH_hs_0025817	0.021	2.106	Catalyzes the hydrolysis of arginine to ornithine and urea
DEFA4	PH_hs_0000344	0.086	2.098	A neutrophil protein thought to be involved in host defense
SLC27A3	PH_hs_0025689	0.039	2.024	Protein with acyl-CoA ligase activity for LCFA and VLCFA
USH1C	PH_hs_0023496	0.086	2.022	May be involved in protein-protein interaction
FBXW12	PH_hs_0035757	0.067	1.893	Substrate-recognition component of SCF-type E3 ubiquitin ligase
CRISP3	PH_hs_0024631	0.027	1.891	A secreted protein found in the salivary gland, pancreas and prostate
USP30	PH_hs_0026074	0.084	1.819	Responsible for c-terminal deubiquitination
ANXA3	PH_hs_0021146	0.039	1.793	Important in cell growth/signaling and possibly anti-coagulation
HIST1H4I	PH_hs_0029514	0.071	1.786	A member of the histone H4 family
PROS1	PH_hs_0003988	0.054	1.751	Helps to prevent coagulation and stimulates fibrinolysis
GYG1	PH_hs_0010438	0.015	1.722	Involved in glycogen anabolism
ANKRD22	PH_hs_0032205	0.063	1.676	Ankyrin repeat domain-containing protein 22
GADD45A	PH_hs_0004630	0.025	1.596	Responds to environmental stresses through activation of p38/JNK
F5	PH_hs_0002589	0.010	1.587	Coagulation factor V which circulates in plasma
KIF15	PH_hs_0023756	0.062	1.577	A member of the kinesin-like protein family
ST14	PH_hs_0003679	0.031	1.551	Degrades extracellular matrix and may play a role in cancer invasion
HIST1H2BG	PH_hs_0034684	0.072	1.546	A member of the histone H2B family
CLU	PH_hs_0025525	0.038	1.545	A secreted protein of unknown function
C19orf59	PH_hs_0010615	0.093	1.523	Speculated to be involved in regulating mast cell differentiation
ATP9A	PH_hs_0019278	0.053	1.512	Catalyzes ATP+H_2_O+phospholipid(In) = ADP+phosphate+phospholipid
FKBP5	PH_hs_0000782	0.046	1.511	Plays a role in immunoregulation, protein folding, and trafficking
ASGR2	PH_hs_0000166	0.092	1.510	Mediates endocytosis of plasma glycoproteins
SLC37A3	PH_hs_0025758	0.025	1.500	Sugar phosphate exchanger 3 (Solute carrier family 37 member 3)

Abbreviations: PC, pancreatic cancer; HC, healthy controls; PBMC, peripheral blood mononuclear cells; FDR, false discovery rate.

**Table 3:** Genes Shown to be at Least 1.5 Fold Downregulated in PBMCs of PC Patients.

*Gene symbol*	*Unique id*	*FDR*	*Fold-change (PC/HC)*	*Gene Information and Normal Gene Function*
				
CCR5	PH_hs_0031237	0.021	0.66	A chemokine receptor expressed by T cells and macrophages
MTAC2D1	PH_hs_0003705	0.058	0.66	Tandem C2 domains
UBASH3A	PH_hs_0010839	0.052	0.66	Promotes accrual of activated TCRs, EGFR and PDGFRB on cell surface
AKT3	PH_hs_0023601	0.013	0.66	An AKT serine/threonine kinase stimulated by PDGF, insulin, and IGF1
PRF1	PH_hs_0000291	0.097	0.65	Perforin, non-specifically lyses target cells
PKIA	PH_hs_0020144	0.014	0.65	Potent competitive inhibitor of cAMP-dependent protein kinase activity
PLEKHA1	PH_hs_0014922	0.058	0.65	Binds specifically to PtdIns3,4P2, highly expressed in the pancreas
AQP3	PH_hs_0012796	0.003	0.65	A water channel protein that also transports of nonionic small solutes
CD1C	PH_hs_0000177	0.027	0.65	Mediates the presentation lipid and glycolipid antigens to T cells
LRRC8C	PH_hs_0002135	0.020	0.64	Leucine-rich repeat-containing protein 8C
GZMA	PH_hs_0005055	0.027	0.64	A T cell and NK cell serine protease, possibly needed for target cell lysis
SH2D1A	PH_hs_0009133	0.008	0.64	An inhibitor of SLAM self-association
PTPN4	PH_hs_0023771	0.010	0.63	Responsible for protein tyrosine dephosphorylation
CD5	PH_hs_0003778	0.028	0.63	May act as a receptor in regulating T-cell proliferation
PTPRCAP/CORO1B	PH_hs_0009399	0.071	0.63	PTPRCAP is a regulator of T- and B-lymphocyte activation.CORO1B regulates leading edge dynamics and cell motility in fibroblasts
FOSB	PH_hs_0002354	0.001	0.63	Dimerizes with JUN proteins to form the AP-1 complex
LCK	PH_hs_0000240	0.009	0.63	Essential to TCR-linked signal transduction and T-cell proliferation
LY9	PH_hs_0024619	0.015	0.63	May participate in adhesion between T lymphocytes and accessory cells
CD3G	PH_hs_0000306	0.095	0.63	CD3-gamma, important in T-cell response to antigen recognition
LEF1	PH_hs_0003252	0.027	0.62	Regulates T-cell receptor alpha enhancer function
CA5B	PH_hs_0039389	0.001	0.62	A zinc metalloenzyme that catalyzes the hydration of carbon dioxide
CD3D	PH_hs_0005206	0.020	0.62	CD3-delta, important in T-cell response to antigen recognition
TRAT1	PH_hs_0031781	0.069	0.61	Stabilizes the TCR/CD3 complex at the surface of T-cells
LAT	PH_hs_0026523	0.019	0.61	Recruits downstream proteins near the site of TCR engagement
KIAA0748	PH_hs_0002788	0.085	0.61	hypothetical protein LOC9840
CD3G	PH_hs_0030882	0.004	0.60	CD3-gamma, important in T-cell response to antigen recognition
GPR115	PH_hs_0022672	0.059	0.60	An orphan G-protein coupled receptor 2
VSIG9	PH_hs_0033271	0.017	0.59	Thought to assist in regulating T-cell dependent B-cell responses
TRAV20/TRDV2	PH_hs_0036583	0.068	0.58	T cell receptor alpha variable 20T cell receptor delta variable 2
CD160	PH_hs_0004672	0.005	0.58	Associates with NK cell and CD8 T-cell cytolytic activity, found on T cells
DYRK2	PH_hs_0010380	0.004	0.57	Activates TP53 to induce apoptosis in response to DNA damage
EBI2	PH_hs_0040414	0.043	0.57	Predicted to encode a GPCR related to the thrombin receptor, found on B cells
MS4A1	PH_hs_0025653	0.015	0.56	Helps in the development and differentiation of B-cells into plasma cells
CCR3	PH_hs_0026576	0.061	0.55	A receptor for C-C type chemokines
EDG1	PH_hs_0009283	0.026	0.54	Possibly involved in regulating endothelial cell differentiation
FCER1A	PH_hs_0000108	0.058	0.53	An IgE receptor found on Mast cells central to allergic disease
EBI2	PH_hs_0000092	0.001	0.52	Predicted to encode a GPCR closely related to the thrombin receptor
GRAMD1C	PH_hs_0037695	0.009	0.52	GRAM domain containing 1C, Function unknown

Abbreviations: PC, pancreatic cancer; HC, healthy controls; PBMC, peripheral blood mononuclear cells; FDR, false discovery rate.

**Figure 1:**
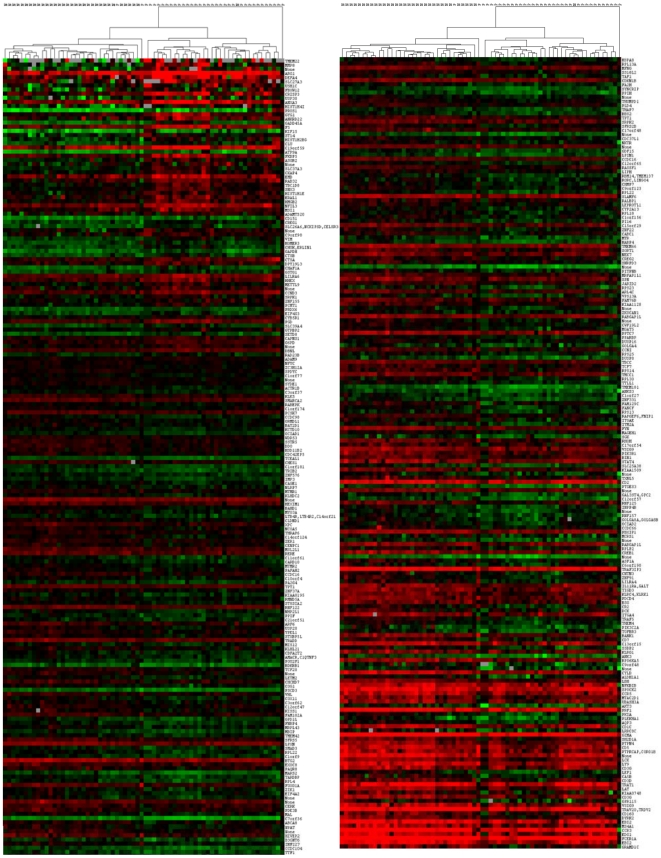
Global gene expression analysis in PBMCs from PC patients and healthy controls. Hierarchical cluster analysis of global gene expression profile by cDNA whole genome microarray comparing healthy control and PC samples using all genes found to be statistically differentially expressed between the two groups (FDR<0.10, n = 383 genes). In no instance were samples pooled. *Red* indicates genes whose expression is elevated relative to the universal human reference (used to normalize all arrays) and *green* indicates genes whose expression is decreased relative to the universal human reference.

**Figure 2:**
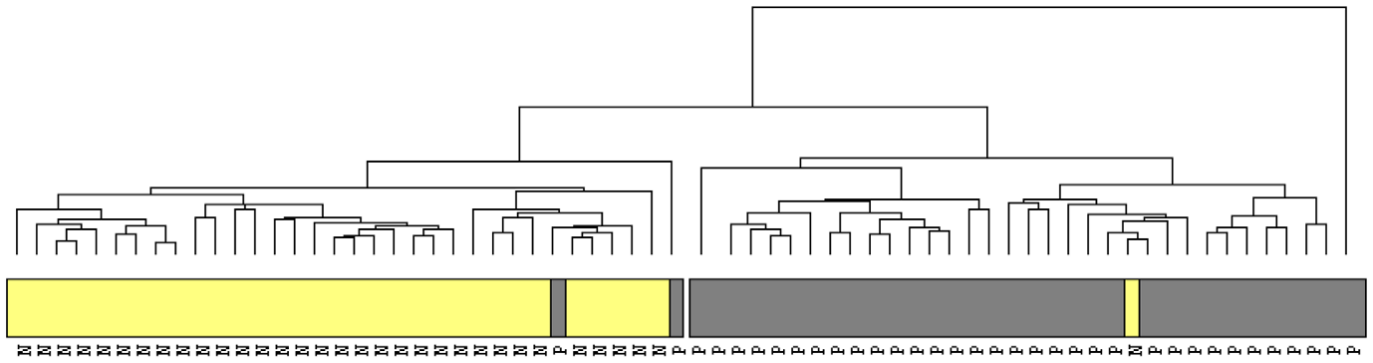
Dendrogram of sample relatedness. A dendrogram of sample relatedness from the cluster analysis shown in Figure 1 using the statistically significant differentially expressed genes. Samples clustered into main groups, aligning well with classification of PC or HC. PC PBMC samples are indicated by *grey bars* while healthy PBMC samples are denoted by *yellow bars*.

A hierarchical clustering of the microarray data identified two clusters of samples, shown in Figure 1 and in dendrogram form in Figure 2, a PC group and a healthy control group. Two PC samples however clustered with the healthy controls, while one healthy control fell into the cluster containing the majority (32/35) of the PC samples. Additionally, the gene expression profile of one PC sample did not cluster with either the healthy controls or the other PC samples.

### Q-RT PCR Validation

Six of the most differentially expressed genes (*ANKRD22, ANXA3, ARG1, FCER1A, GRAMD1C*, and *MS4A1*) were chosen for validation by Q-RT PCR in a randomly selected subset of 21 PBMC samples (comprised of 12 PC samples and 9 healthy control samples from the original 68 used in the microarray). The median fold expression for five of them was in the same direction as that in the microarray, giving us a validation rate of 83%. *FCER1A* was the only gene for which a positive correlation was not obtained. The results are depicted in Table 4.

**Table 4:** Median fold change (PDAC/Normal) of selected genes chosen for validation by Q-RT PCR.

*Gene Name*	*Microarray*	*Q-RT PCR*
ANKRD22	1.68	1.16
ANXA3	1.79	1.71
ARG1	2.11	1.95
FCER1A	0.53	1.24
GRAMD1C	0.52	0.49
MS4A1	0.56	0.62

Microarray results were validated by quantitative real time PCR (Q-RT PCR) using SYBR green based chemistry. 3 genes shown to be up-regulated by microarray (*ANKRD22, ANXA3, ARG1)* and 3 down-regulated *(FCER1A, GRAMD1C, and MS4A1*) genes were validated in a randomly selected subset of samples that included 9 healthy controls and 12 PC patients. The fold-change in gene expression was determined by the 2^-ΔΔCt^ method using the same human reference RNA as the standard employed in the microarray. Correlation between microarray and Q-RT PCR results were determined through calculation of the median fold change for the PDAC and healthy samples by Q-RT PCR and comparing it to the fold change seen by microarray to determine whether the gene was differentially expressed in the same direction using both methods.

### Correlation of PBMC expression profile with clinicopathologic characteristics

To determine whether a correlation existed between the PBMC gene expression profile in PC patients and clinically relevant patient characteristics, we divided the PC samples based on surgical status (23 pre-surgery vs. 12 post-surgery), history of chemotherapy (15 pre-chemotherapy vs. 20 post-chemotherapy), diagnosis of type-II diabetes mellitus prior to the diagnosis of PC (14 with a positive history vs. 19 with a negative history), location of the primary tumor (25 head vs. 8 body/tail), and stage of the PC at diagnosis (6 localized (Stage 1/2A) vs. 12 non-localized non-metastatic (Stage 2B/3) vs. 15 metastatic (Stage 4) PC). However, we did not observe any significant difference in gene expression between any of these patient groups applying the criterion of an FDR <10%.

### Gene Predictor Set

We next investigated whether we could identify a minimal gene-predictor set that would accurately discriminate PC cases from healthy controls. To do this, 44/68 samples comprising 24 PC and 20 healthy control samples were randomly chosen. All 21,671 genes for each of the samples were entered into the analysis. An eight-gene predictor set was obtained and comprised of *SSBP2, Ube2b-rs1, CA5B, F5, TBC1D8, ANXA3, ARG1*, and *ADAMTS20*. Using the Compound Covariate Prediction Method (CCPM), this predictor set gave a correct classification of PC vs. non-PC with an accuracy of 73%, providing a sensitivity of 71% and a specificity of 75%. The weights given to each gene using CCPM were –4.97, –4.83, –4.38, 4.43, 4.44, 4.53, 4.84, and 4.96 respectively with a threshold value of 38.98 such that if Σ_i_ *w*_i_ *x*_i_ was > the threshold for a sample it was predicted as being from a PC patient (where *w*_i_ =  gene weight, *x*_i_ =  log_2_ gene expression intensity).

### Validation of Gene Predictor Set

Using this eight-gene predictor set, classification of a sample as being either PC or a healthy control was attempted in a blinded manner using a sample set consisting *only* of the samples that were not used to create the predictor set (i.e. 24/68). In this blinded validation, using the equation derived above, the gene predictor set accurately predicted the diagnosis of PC with 73% accuracy, giving a sensitivity of 83% and a specificity of 64%.

In an attempt to further test the potential diagnostic utility of this gene predictor set, a new subset of samples, comprising of 12 PC samples obtained from patients who were both pre-chemotherapy and pre-surgery, along with an equal number of randomly selected healthy controls, were again blinded and analyzed to predict their classification. This time the eight-gene predictor was able to correctly classify these samples 79% of the time, giving an 83% sensitivity and 75% specificity of diagnosis.

## Discussion

In recent years it has been repeatedly demonstrated that genetic expression in PBMCs is altered in the context of malignancy [13], [14], [21], [22]. This observation of an altered PBMC genetic expression profile in cancer patients was first reported in diffuse large B-cell lymphoma and chronic lymphocytic leukemia and later extended beyond hematological malignancies through the analysis of PBMC expression profiling in patients with advanced renal cell carcinoma (RCC) [13]–[14]. In both hematologic malignancies and in RCC, it was reported that the variation in gene expression between patients with disease and healthy controls was much greater than the inter-sample variation observed for the healthy patients alone, suggesting that PBMCs could be useful surrogate markers with potential diagnostic and prognostic applications in cancer. Further, in RCC, it was shown that an 8-gene classifier set developed from the differentially expressed genes could predict the diagnosis of malignancy with 100% accuracy [14].

Recently, Huang et al. have reported that a differential gene expression profile does exist in PBMCs of PC patients [22]. While this study also used microarray and Q-RT PCR validation to establish differential genetic expression in the peripheral blood of PC patients, its purpose was to establish potential biomarkers that could differentiate newly diagnosed diabetic patients with PC from diabetics without PC. While the study authors reported that 48 genes were differentially expressed between PC patients and healthy controls by microarray, only 8 samples were used in each of the two groups and they provided no further information regarding these genes. The smaller sample size and a lack of blinded validation further contrast this study with the present report. Additionally, we did not find any significantly differentially expressed genes based on history of either prior surgery or chemotherapy, history of type-II diabetes mellitus, or stage of PC in our study. Importantly, the study by Huang et al. utilized *GAPDH* as the housekeeping gene against which the expression of every gene was normalized. In our study, however, we noted that *GADPH* was one of the most significantly overexpressed genes in PBMCs of PC patients. Upregulation of *GAPDH* has also been reported in several malignancies including ovarian, thyroid, hepatocellular and pancreatic cancers [23]–[26]. The choice of the ideal internal reference gene in studies investigating potential clinical biomarkers by microarray remains an important question that will need to be addressed in future studies.

**Figure 3:**
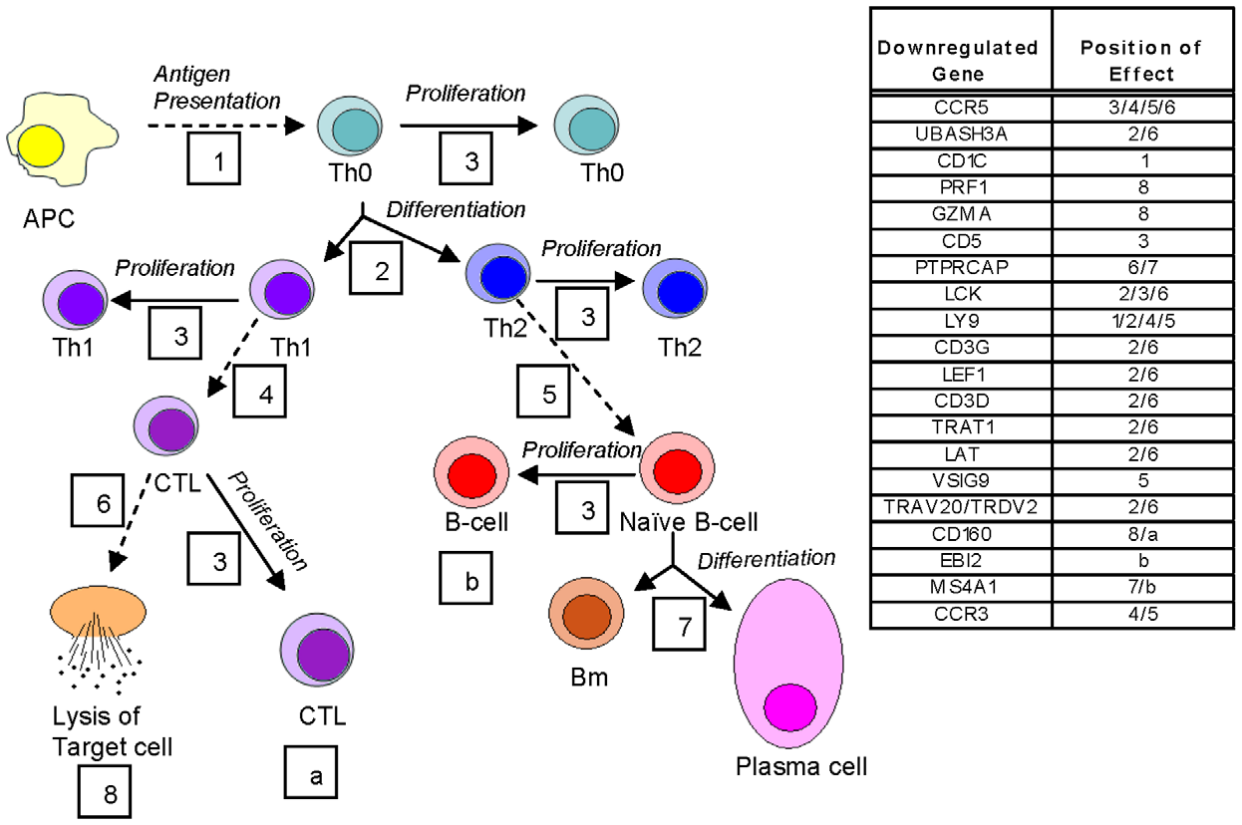
Potential effect of the differential genetic expression of PBMCs. All genes shown were found to be down-regulated greater than 1.5 fold. The respective amount of differential expression per gene as well as the stated function can be found in Table 3. The differential expression of these genes indicate that there is a global decrease in cell number, activation, and effectiveness of the adaptive immune system in patients with PDAC that may have a significant effect on both their morbidity and mortality. Dashed lines indicate the association of cells while solid lines indicate the differentiation or proliferation of a particular cell type. Numbers represent individual points of interaction between the genes and the immune differentiation and response pathway: **1**, Presentation of antigen to Th0 cells; **2**, Differentiation of Th0 cells down the Th1 or Th2 pathway; **3**, Immune cell proliferation; **4**, Stimulation of cytotoxic T-cell activity by Th1 cells; **5**, Stimulation of humoral immunity by Th2 cells; **6**, Recognition and response to target cells by cytotoxic T lymphocytes (CTL); **7**, Differentiation of naïve B-cells; **8**, Lysis of target cells by CTLs. Letters represent individual cell populations: **a**, Cytotoxic T-lymphocytes (CTL); **b**, B-cells. Decrease in genes associated with points **a** and **b** may represent a decrease in their respective associated cell's population.

This present study represents the first in-depth analysis of the transcriptome of PBMCs from patients with PC compared to healthy controls, and only the third instance of such profiling for solid tumors in general. Establishment of such differential expression has the potential to yield a rich compendium of potential genes for further pursuit as novel diagnostic or therapeutic targets. Further, the gene networks identified in our study offer novel insights into the disregulation of the immune system in PC (Figure 3). With the fact that only 15–20% of PC patients are diagnosed with resectable disease and given the stubborn resistance of the malignancy to chemo and radiotherapy, early detection of the disease offers the greatest hope for an immediate impact on improving patient prognosis.

The potential for PBMC differential gene expression profiling, or of a pre-determined gene predictor set established from it, to be useful for early diagnosis of PC is theoretically quite high; especially when it is considered that the two most likely mechanisms underlying this differential expression are the immune system's recognition of the cancer and the evasion of the immune system by the cancer. While other biomarkers, such as CA19-9, are released from the cancer cells and thus rise with increasing tumor burden, differential expression in PBMCs may begin, at least partially, as soon as cancer immunogenicity or immune evasion is established. Immune system evasion has been shown to be initiated as early as pre-malignant disease in PC, thus supporting the premise that analysis of differential gene expression in immune cells may offer the ability to detect a neoplastic lesion even before it gains invasive capabilities [27].

While this study itself does not attempt to look at the early diagnostic capabilities of PBMCs, the results obtained from the work are a necessary first step towards a multiplexed assay based on alteration of gene expression in PC for potential application in high-risk groups [28]. The fact that an 8-gene predictor set was able to establish a sensitivity of 83% with a specificity of between 64 and 75% in a blinded set of samples is promising and will need to be validated in a large sample set. While the number of samples is too small to perform any further detailed analysis, the fact that the sensitivity for the gene predictor set did not decrease when applied to PC patients prior to chemotherapy or surgery points toward the potential utility of this 8-gene predictor set in a diagnostic setting, the main area in which CA19-9 is lacking [4]–[8]. Additionally, PBMC gene expression analysis is no more invasive of a test than CA19-9, both being amenable to a simple venopuncture, and the overall analysis need not be substantially more expensive than current clinical methods for testing CA19-9. If only the 8-gene classifier set is used for analysis, PBMC testing could be accomplished through the use of mini-cDNA microarray chips or through multiplex PCR reactions, both of which are clinically viable and would be fairly simple to add to the repertoire of tests provided by a standard clinical lab.

Beyond the diagnostic potential of this PBMC differential expression profile, the normal functions and direction of differential expression of each of the genes, especially the 65 that were ≥1.5 fold differentially expressed, hints at potential pathophysiologic mechanisms. 18/65 genes have the potential to directly decrease T-cell proliferation, T-cell receptor signaling, or cytotoxic T-lymphocyte (CTL) cytotoxicity while four can directly modulate a decrease in B-cell activation/differentiation or signal a decrease in the number of circulating B-cells. Three of the genes can directly decrease the cytotoxicity of NK cells, and two can decrease macrophage response. Taken together, the results of our study suggest that PC is characterized by a significant decrease in the ability of the immune system to respond to non-self antigens, including tumor associated antigens, as summarized in Figure 3. A partial hint about the mechanisms underlying this immune compromise may come from the observed upregulation of *ARG1*, observed to be upregulated more than 2-fold in PBMCs of PC patients. An expression of ARG1 is closely associated with an increase in the presence of myeloid derived suppressor cells (MDSCs) [29]. MDSCs are classically known to decrease CTL response, mostly through destabilization of T-cell receptors and decreased expression of certain CD3 subtypes, ultimately leading to CTL apoptosis. However, MDSCs are known to specifically cause the down regulation of CD3Z, which was not shown to be differentially expressed in PBMCs analyzed in this study [29]. Additionally, MDSCs are known to cause a funneling of the immune system away from cellular immunity and toward humoral and allergic-response immunity, a property that is not clearly represented in the PBMC differential expression data. Conversely, it appears (from the alteration in gene expression) that the number of circulating B-cells is decreasing while both *FCER1A*, a receptor central to allergic response, and *MS4A1*, which plays a role in B-cell to plasma cell differentiation, are down regulated. Thus, while MDSCs may play a role in modulating the differential expression seen in PBMCs of PC patients, they likely operate in concert with other mechanisms to affect a down-regulation of both the body's cellular and humoral immune response machinery.

A comparison of the gene expression profile observed in our study with that reported in other diseases revealed little similarity with other benign (pre-eclampsia, rheumatoid arthritis (RA), and chronic pancreatitis (CP)) and malignant diseases (RCC). In total, 6 genes (*CD160, GOLGA8B, RABGAP1L, MMP8, CRISP3*, and *ARG1*) that were shown to be differentially expressed in PBMCs of patients with pre-eclampsia were also differentially expressed in PBMCs of PC patients, with 4 (*CD160, MMP8, CRISP3*, and *ARG1*) being differentially expressed in the same direction (1% commonality) [10]. Twelve genes (*BTG2, CCND3, CD151, CD7, CLU, CTSB, KLRK1, SPN, GSTO1, PCMT1, PRDX6*, and *PRF1*) that were shown to be differentially expressed in PBMCs of patients with RA were also differentially expressed in PBMCs of PC patients, 8 of which (*CCND3, CD151, CLU, CTSB, GSTO1, PCMT1, PRDX6*, and *PRF1*) were in the same direction (2% commonality) [11]. Two genes (PDE3B and GADD45a) found to be differentially expressed in PBMCs of CP patients were also differentially expressed in PBMCs of PC patients, neither of which being differentially expressed in the same direction (0% commonality) [12]. However, there was no similarity in the list of significantly differentially expressed genes between PC and RCC [14]. These results strongly suggest that the gene expression observed in the present study is highly specific to PC, and thus increases the potential applicability of differential expression profiling of PBMCs as a diagnostic tool in PC.

To investigate the possibility that the gene expression profile in PBMCs is a reflection of the genes expressed in the PC tissues itself, we also investigated if there was a similarity between the differentially expressed genes identified in our study and those identified by microarray analysis in pancreatic primary tumors. In total it was found that, of the 383 differentially expressed genes found in PBMCs of PC patients, only 4 (*ADAM9, IMP3, BTG2*, and *G6PD*) were also shown to be differentially expressed in PC primary tissues, with 2 (*ADAM9* and *BTG2*) being differentially expressed in the same direction (0.5% commonality) [30]. Thus it appears that, in general, the gene expression in PBMCs does not mimic that in the primary tumor.

Further, if circulating tumor cells from the pancreas were the cause of the differential expression profile seen in this study, it would be expected that genes normally expressed in pancreatic cells but not peripheral blood cells would be identified by microarray. While this was the case for *USH1C, CRISP3*, and *USP30*, all genes that are expressed at low to moderate levels in the pancreas but not expressed normally in the peripheral blood, *PLEKHA1*, a gene that is normally highly expressed in the pancreas but only expressed at very low levels in the peripheral blood, was shown to be down regulated in our samples, adding to the evidence that the differential expression we report truly is from PBMCs.

In conclusion, we have shown that a differential gene expression profile exists in PBMCs of patients with PC. Further, an 8 gene classifier set has been established which provides, in a blinded subset of our samples, an improved sensitivity over CA19-9 with a similar specificity [3], [4], [6], [28]. Significantly, there was no decrease in sensitivity when employing samples from patients prior to any form of chemotherapy or surgery. Comparison with other studies points toward this differential expression profile as being specific to PBMCs and particularly to PC. Additionally, the differential gene expression seems to represent a systemic compromise of both cellular and humoral immunity, although it does not point toward one particular underlying mechanism.

Based on these results, future research is needed to establish the various mechanisms behind PC-induced differential PBMC genetic expression and how much effect this differential expression actually has on the body's immunologic capabilities. Further, the 8 gene classifier set must be tested in an expanded set of both healthy controls and PC patients as well as in a set of non-PC patients with benign/malignant disease to clarify its sensitivity and specificity. PC sample selection for such a study should be biased toward early stage patients to elicit the diagnostic capabilities of PBMC differential expression in the patient population in which it has the greatest likelihood of having a positive impact on patient outcome. Due to the difficulty of attaining ample specimens from early stage patients, preliminary study of early stage PC diagnosis through PBMC expression analysis may be first carried out in a spontaneous PC murine model which recapitulates the preneoplastic and early neoplastic processes seen in human PC as a proof of concept [31], [32]. Upon conclusion of such a murine study, resources could then be expended in the recruitment and testing of enough early stage human PC subjects for ample analysis to be conducted. Once PBMC expression has been diagnostically validated in an expanded sample set, the gene set could also be used to characterize potential prognostic abilities of the PBMC differential expression in PC.

Though further studies are necessary to contingently state the diagnostic and prognostic potential of PBMC gene expression profiling in general and of the 8 gene PC classifier set in particular, our current results are promising and point toward the potential for peripheral blood mononuclear cells to be highly efficacious tools for improving the prognosis of one of the world's deadliest cancers.

## Supporting Information

Table S1 **Genes Shown to be Statistically Differentially Expressed in PBMCs of PDAC Patients (FDR<0.10).** Global expression profiles of peripheral blood mononuclear cells from 26 pancreatic ductal adenocarcinoma (PC) patients and 33 age, race, and gender matched healthy controls were compared by whole genome microarray. After normalization, filtering, and statistical analysis, 383 genes were found to be significantly differentially expressed (FDR<0.10) between the two groups.
(DOC)

## References

[1] The American Cancer Society: Cancer Facts and Figures (2009). http://www.cancer.org/Research/CancerFactsFigures/index.

[2] National Cancer Institute: Surveillance Epidemiology and End Results Cancer Statistics Review (1975-2006). http://seer.cancer.gov/csr/1975_2006/index.html.

[3] Ni XG, Bai XF, Mao YL, Shao YF, Wu JX (2005). The clinical value of serum CEA, CA19-9, and CA242 in the diagnosis and prognosis of pancreatic cancer.. Eur J Surg Oncol.

[4] (1999). Tumour markers in gastrointestinal cancers—EGTM recommendations. European Group on Tumour Markers.. Anticancer Res.

[5] DiMagno EP, Reber HA, Tempero MA (1999). AGA technical review on the epidemiology, diagnosis, and treatment of pancreatic ductal adenocarcinoma. American Gastroenterological Association.. Gastroenterology.

[6] Goggins M (2005). Molecular markers of early pancreatic cancer.. J Clin Oncol.

[7] Pleskow DK, Berger HJ, Gyves J, Allen E, McLean A (1989). Evaluation of a serologic marker, CA19-9, in the diagnosis of pancreatic cancer.. Ann Intern Med.

[8] Steinberg W (1990). The clinical utility of the CA 19-9 tumor-associated antigen.. Am J Gastroenterol.

[9] Liang JJ, Kimchi ET, Staveley-O'Carroll KF, Tan D (2009). Diagnostic and prognostic biomarkers in pancreatic carcinoma.. Int J Clin Exp Pathol.

[10] Sun CJ, Zhang L, Zhang WY (2009). Gene expression profiling of maternal blood in early onset severe preeclampsia: identification of novel biomarkers.. J Perinat Med.

[11] Edwards CJ, Feldman JL, Beech J, Shields KM, Stover JA (2007). Molecular profile of peripheral blood mononuclear cells from patients with rheumatoid arthritis.. Mol Med.

[12] Bluth M, Lin Y, Zhang H, Viterbo D, Zenilman M (2008). Gene expression profiles in cells of peripheral blood identify new molecular markers of acute pancreatitis.. Arch Surg.

[13] Whitney AR, Diehn M, Popper SJ, Alizadeh AA, Boldrick JC (2003). Individuality and variation in gene expression patterns in human blood.. Proc Natl Acad Sci U S A.

[14] Twine NC, Stover JA, Marshall B, Dukart G, Hidalgo M (2003). Disease-associated expression profiles in peripheral blood mononuclear cells from patients with advanced renal cell carcinoma.. Cancer Res.

[15] Hedenfalk I, Duggan D, Chen Y, Radmacher M, Bittner M (2001). Gene-expression profiles in hereditary breast cancer.. N Engl J Med.

[16] Chang JC, Wooten EC, Tsimelzon A, Hilsenbeck SG, Gutierrez MC (2003). Gene expression profiling for the prediction of therapeutic response to docetaxel in patients with breast cancer.. Lancet.

[17] Hu N, Wang C, Hu Y, Yang HH, Giffen C (2005). Genome-wide association study in esophageal cancer using GeneChip mapping 10K array.. Cancer Res.

[18] Ramaswamy S, Tamayo P, Rifkin R, Mukherjee S, Yeang CH (2001). Multiclass cancer diagnosis using tumor gene expression signatures.. Proc Natl Acad Sci U S A.

[19] Torres MP, Ponnusamy MP, Chakraborty S, Smith LM, Das S (2010). Effects of thymoquinone in the expression of mucin 4 in pancreatic cancer cells: implications for the development of novel cancer therapies.. Mol Cancer Ther.

[20] Simon R, Lam A, Li MC, Ngan M, Menenzes S (2007). Analysis of Gene Expression Data Using BRB-Array Tools.. Cancer Inform.

[21] Burczynski ME, Twine NC, Dukart G, Marshall B, Hidalgo M (2005). Transcriptional profiles in peripheral blood mononuclear cells prognostic of clinical outcomes in patients with advanced renal cell carcinoma.. Clin Cancer Res.

[22] Huang H, Dong X, Kang MX, Xu B, Chen Y (2010). Novel Blood Biomarkers of Pancreatic Cancer-Associated Diabetes Mellitus Identified by Peripheral Blood-Based Gene Expression Profiles.. Am J Gastroenterol.

[23] Hansen CN, Ketabi Z, Rosenstierne MW, Palle C, Boesen HC (2009). Expression of CPEB, GAPDH and U6snRNA in cervical and ovarian tissue during cancer development.. APMIS.

[24] Giusti L, Iacconi P, Ciregia F, Giannaccini G, Donatini DL (2008). Fine-needle aspiration of thyroid nodules: proteomic analysis to identify cancer biomarkers.. J Proteome Res.

[25] Waxman S, Wurmbach E (2007). De-regulation of common housekeeping genes in hepatocellular carcinoma.. BMC Genomics.

[26] Mikuriya K, Kuramitsu Y, Ryozawa S, Fujimoto M, Mori S (2007). Expression of glycolytic enzymes is increased in pancreatic cancerous tissues as evidenced by proteomic profiling by two-dimensional electrophoresis and liquid chromatography-mass spectrometry/mass spectrometry.. Int J Oncol.

[27] Zhao F, Obermann S, von WR, Haile L, Manns MP (2009). Increase in frequency of myeloid-derived suppressor cells in mice with spontaneous pancreatic carcinoma.. Immunology.

[28] Chakraborty S, Baine MJ, Sasson AR, Batra SK (2010). Current status of molecular markers for early detection of sporadic pancreatic cancer.. Biochim Biophys Acta.

[29] Talmadge JE (2007). Pathways mediating the expansion and immunosuppressive activity of myeloid-derived suppressor cells and their relevance to cancer therapy.. Clin Cancer Res.

[30] Brandt R, Grutzmann R, Bauer A, Jesnowski R, Ringel J (2004). DNA microarray analysis of pancreatic malignancies.. Pancreatology.

[31] Frese KK, Tuveson DA (2007). Maximizing mouse cancer models.. Nat Rev Cancer.

[32] Hingorani SR, Petricoin EF, Maitra A, Rajapakse V, King C (2003). Preinvasive and invasive ductal pancreatic cancer and its early detection in the mouse.. Cancer Cell.

