# Supplementary material for: Transcriptional Profiling of Peripheral Blood Mononuclear Cells in Pancreatic Cancer Patients Identifies Novel Genes with Potential Diagnostic Utility
Source: PLoS One. 2011 Feb 10;6(2):e17014. doi: 10.1371/journal.pone.0017014 (PMC3037404; doi:10.1371/journal.pone.0017014)
Supplement: Table S1 — Genes Shown to be Statistically Differentially Expressed in PBMCs of PDAC Patients (FDR<0.10). Global expression profiles of peripheral blood mononuclear cells from 26 pancreatic ductal adenocarcinoma (PC) patients and 33 age, race, and gender matched healthy controls were compared by whole genome microarray. After normalization, filtering, and statistical analysis, 383 genes were found to be significantly differentially expressed (FDR<0.10) between the two groups. (DOC) [file pone.0017014.s001.doc]

| **Table S1: Genes Shown to be Statistically Differentially Expressed in PBMCs of PDAC Patients (FDR<0.10)** | | | | | |
| --- | --- | --- | --- | --- | --- |
| *Gene symbol* | *Unique id* | *FDR* | *Geom mean of ratios in Cancer group* | *Geom mean of ratios in Normal group* | *Fold-change (Cancer/Normal)* |
| TMEM22 | PH_hs_0038059 | 0.0687 | 2.777 | 0.577 | 4.812 |
| MMP8 | PH_hs_0024515 | 0.0763 | 1.776 | 0.755 | 2.351 |
| None | PH_hs_0040408 | 0.0429 | 1.296 | 0.592 | 2.188 |
| ARG1 | PH_hs_0025817 | 0.0211 | 11.461 | 5.443 | 2.106 |
| DEFA4 | PH_hs_0000344 | 0.0862 | 3.941 | 1.879 | 2.098 |
| SLC27A3 | PH_hs_0025689 | 0.0389 | 2.783 | 1.375 | 2.024 |
| USH1C | PH_hs_0023496 | 0.0862 | 1.738 | 0.860 | 2.022 |
| FBXW12 | PH_hs_0035757 | 0.0669 | 1.198 | 0.633 | 1.893 |
| CRISP3 | PH_hs_0024631 | 0.0273 | 3.371 | 1.782 | 1.891 |
| USP30 | PH_hs_0026074 | 0.084 | 0.948 | 0.521 | 1.819 |
| ANXA3 | PH_hs_0021146 | 0.0385 | 5.152 | 2.874 | 1.793 |
| HIST1H4I | PH_hs_0029514 | 0.0712 | 1.189 | 0.666 | 1.786 |
| PROS1 | PH_hs_0003988 | 0.0541 | 0.362 | 0.207 | 1.751 |
| GYG1 | PH_hs_0010438 | 0.0154 | 2.246 | 1.304 | 1.722 |
| ANKRD22 | PH_hs_0032205 | 0.063 | 2.132 | 1.272 | 1.676 |
| GADD45A | PH_hs_0004630 | 0.0253 | 0.663 | 0.416 | 1.596 |
| F5 | PH_hs_0002589 | 0.0105 | 3.552 | 2.239 | 1.587 |
| KIF15 | PH_hs_0023756 | 0.0623 | 0.410 | 0.260 | 1.577 |
| ST14 | PH_hs_0003679 | 0.0308 | 1.168 | 0.753 | 1.551 |
| HIST1H2BG | PH_hs_0034684 | 0.0719 | 1.132 | 0.732 | 1.546 |
| CLU | PH_hs_0025525 | 0.0375 | 0.854 | 0.553 | 1.545 |
| C19orf59 | PH_hs_0010615 | 0.0926 | 5.530 | 3.632 | 1.523 |
| ATP9A | PH_hs_0019278 | 0.0531 | 0.474 | 0.314 | 1.512 |
| FKBP5 | PH_hs_0000782 | 0.0463 | 2.622 | 1.736 | 1.511 |
| ASGR2 | PH_hs_0000166 | 0.0919 | 1.273 | 0.843 | 1.510 |
| None | PH_hs_0031439 | 0.0541 | 1.364 | 0.905 | 1.506 |
| SLC37A3 | PH_hs_0025758 | 0.0253 | 1.192 | 0.795 | 1.500 |
| CKAP4 | PH_hs_0004243 | 0.0095 | 1.385 | 0.925 | 1.497 |
| EMB | PH_hs_0040245 | 0.0897 | 3.282 | 2.215 | 1.481 |
| RAB32 | PH_hs_0002729 | 0.0495 | 1.958 | 1.338 | 1.464 |
| TBC1D8 | PH_hs_0025860 | 0.0226 | 1.419 | 0.983 | 1.443 |
| SNX3 | PH_hs_0001136 | 0.0041 | 1.451 | 1.008 | 1.440 |
| HIST1H1E | PH_hs_0013158 | 0.0308 | 1.494 | 1.038 | 1.439 |
| ERAL1 | PH_hs_0030897 | 0.0602 | 1.808 | 1.277 | 1.416 |
| HMGB2 | PH_hs_0028423 | 0.0531 | 1.511 | 1.069 | 1.414 |
| NFIL3 | PH_hs_0004587 | 0.0591 | 3.280 | 2.335 | 1.405 |
| MSI1 | PH_hs_0009587 | 0.0866 | 1.974 | 1.424 | 1.386 |
| ADAMTS20 | PH_hs_0014873 | 0.0806 | 1.378 | 1.004 | 1.372 |
| CD151 | PH_hs_0026906 | 0.0712 | 0.495 | 0.361 | 1.369 |
| CREG1 | PH_hs_0030922 | 0.052 | 0.840 | 0.621 | 1.353 |
| SLC26A6,NCKIPSD,CELSR3 | PH_hs_0020493 | 0.0253 | 0.720 | 0.533 | 1.352 |
| None | PH_hs_0039951 | 0.0897 | 1.363 | 1.012 | 1.347 |
| C9orf98 | PH_hs_0019730 | 0.0742 | 1.447 | 1.086 | 1.332 |
| VIM | PH_hs_0025444 | 0.0712 | 0.889 | 0.668 | 1.331 |
| HOMER3 | PH_hs_0024838 | 0.0384 | 0.756 | 0.569 | 1.328 |
| CHUK,ERLIN1 | PH_hs_0032500 | 0.0211 | 0.804 | 0.606 | 1.326 |
| GAPDH | PH_hs_0037816 | 0.063 | 0.551 | 0.416 | 1.324 |
| CTSB | PH_hs_0031766 | 0.0954 | 0.749 | 0.568 | 1.319 |
| CTSA | PH_hs_0006486 | 0.0669 | 2.001 | 1.520 | 1.316 |
| DPY19L3 | PH_hs_0035677 | 0.0799 | 1.617 | 1.239 | 1.304 |
| CHAF1A | PH_hs_0033183 | 0.0374 | 0.612 | 0.470 | 1.301 |
| GSTO1 | PH_hs_0028760 | 0.0372 | 0.757 | 0.582 | 1.300 |
| LILRA6 | PH_hs_0026618 | 0.0763 | 2.484 | 1.918 | 1.295 |
| HHEX | PH_hs_0006542 | 0.0588 | 2.129 | 1.645 | 1.295 |
| METTL9 | PH_hs_0014366 | 0.0914 | 1.186 | 0.919 | 1.290 |
| None | PH_hs_0036161 | 0.0954 | 1.499 | 1.165 | 1.287 |
| CCND3 | PH_hs_0004752 | 0.0425 | 2.305 | 1.794 | 1.284 |
| SRPK1 | PH_hs_0024102 | 0.0614 | 1.111 | 0.878 | 1.265 |
| ZNF155 | PH_hs_0040876 | 0.091 | 1.246 | 0.986 | 1.264 |
| PCMT1 | PH_hs_0004574 | 0.0359 | 1.162 | 0.925 | 1.256 |
| PRDX6 | PH_hs_0000020 | 0.0927 | 0.624 | 0.502 | 1.243 |
| EIF4G3 | PH_hs_0024612 | 0.092 | 0.732 | 0.589 | 1.242 |
| CYB5R1 | PH_hs_0023860 | 0.0548 | 1.255 | 1.016 | 1.235 |
| PGD | PH_hs_0027429 | 0.0938 | 2.216 | 1.797 | 1.233 |
| SLC39A4 | PH_hs_0035924 | 0.0558 | 0.715 | 0.585 | 1.222 |
| GTPBP2 | PH_hs_0001250 | 0.092 | 1.176 | 0.971 | 1.211 |
| SETD8 | PH_hs_0032504 | 0.0873 | 0.624 | 0.516 | 1.211 |
| CAPNS1 | PH_hs_0033087 | 0.052 | 1.458 | 1.220 | 1.195 |
| G6PD | PH_hs_0004621 | 0.0558 | 1.588 | 1.334 | 1.191 |
| None | PH_hs_0019601 | 0.0976 | 1.332 | 1.121 | 1.188 |
| DBNL | PH_hs_0010817 | 0.0503 | 1.940 | 1.643 | 1.181 |
| RAD23B | PH_hs_0031911 | 0.0794 | 0.812 | 0.690 | 1.177 |
| ADAM9 | PH_hs_0040856 | 0.0928 | 1.234 | 1.120 | 1.102 |
| NFYC | PH_hs_0039272 | 0.0712 | 1.075 | 1.187 | 0.906 |
| ZC3H12A | PH_hs_0014957 | 0.0603 | 1.012 | 1.125 | 0.900 |
| SPDYC | PH_hs_0033769 | 0.0614 | 1.089 | 1.220 | 0.892 |
| C1orf77 | PH_hs_0016120 | 0.0429 | 0.895 | 1.005 | 0.890 |
| None | PH_hs_0022155 | 0.0777 | 1.032 | 1.161 | 0.889 |
| SYDE1 | PH_hs_0000970 | 0.0253 | 1.060 | 1.195 | 0.887 |
| ACTR1B | PH_hs_0000331 | 0.0873 | 0.871 | 0.982 | 0.886 |
| C3orf37 | PH_hs_0033907 | 0.0323 | 0.782 | 0.887 | 0.881 |
| KLK5 | PH_hs_0003518 | 0.0865 | 1.295 | 1.477 | 0.877 |
| SMARCA2 | PH_hs_0033106 | 0.0943 | 1.717 | 1.958 | 0.877 |
| RABEPK | PH_hs_0035376 | 0.0296 | 0.920 | 1.051 | 0.875 |
| C1orf174 | PH_hs_0022255 | 0.0273 | 1.015 | 1.162 | 0.874 |
| PCSK7 | PH_hs_0025922 | 0.0919 | 1.639 | 1.876 | 0.874 |
| CCDC98 | PH_hs_0016473 | 0.0956 | 0.769 | 0.881 | 0.873 |
| ORMDL1 | PH_hs_0026763 | 0.0479 | 0.941 | 1.078 | 0.873 |
| BAT2D1 | PH_hs_0030308 | 0.0954 | 0.950 | 1.090 | 0.872 |
| KCTD10 | PH_hs_0015637 | 0.0273 | 0.800 | 0.919 | 0.870 |
| OCIAD1 | PH_hs_0022807 | 0.0138 | 0.674 | 0.777 | 0.868 |
| WDR53 | PH_hs_0033067 | 0.0127 | 1.141 | 1.320 | 0.864 |
| SSTR5 | PH_hs_0029475 | 0.0554 | 1.216 | 1.408 | 0.864 |
| DDO | PH_hs_0010447 | 0.0584 | 1.180 | 1.367 | 0.863 |
| HSD11B2 | PH_hs_0039052 | 0.0954 | 1.119 | 1.298 | 0.862 |
| CDC42EP5 | PH_hs_0028374 | 0.0954 | 1.047 | 1.217 | 0.860 |
| TCEAL1 | PH_hs_0005202 | 0.0763 | 0.492 | 0.573 | 0.860 |
| CHES1 | PH_hs_0012356 | 0.0256 | 1.280 | 1.489 | 0.860 |
| C1orf181 | PH_hs_0000525 | 0.0851 | 0.621 | 0.723 | 0.859 |
| TRIB2 | PH_hs_0009360 | 0.0901 | 0.570 | 0.665 | 0.858 |
| ZNF576 | PH_hs_0001085 | 0.027 | 0.749 | 0.874 | 0.857 |
| IMP3 | PH_hs_0026912 | 0.0409 | 0.656 | 0.767 | 0.856 |
| CAGE1 | PH_hs_0030001 | 0.0632 | 0.833 | 0.974 | 0.855 |
| NLRP7 | PH_hs_0016956 | 0.0961 | 0.834 | 0.975 | 0.855 |
| MTMR1 | PH_hs_0016735 | 0.0832 | 1.115 | 1.305 | 0.855 |
| KLHDC2 | PH_hs_0025008 | 0.0712 | 0.822 | 0.962 | 0.855 |
| None | PH_hs_0008262 | 0.0984 | 2.453 | 2.873 | 0.854 |
| HEXIM1 | PH_hs_0001428 | 0.0954 | 1.579 | 1.852 | 0.852 |
| BAHD1 | PH_hs_0001925 | 0.0092 | 0.930 | 1.092 | 0.852 |
| MYO3A | PH_hs_0022643 | 0.0797 | 0.829 | 0.974 | 0.852 |
| LTB4R,LTB4R2,C14orf21 | PH_hs_0037920 | 0.0954 | 0.717 | 0.842 | 0.852 |
| CLDND1 | PH_hs_0005965 | 0.0354 | 0.978 | 1.149 | 0.851 |
| XPC | PH_hs_0030850 | 0.0667 | 1.388 | 1.631 | 0.851 |
| NCOA5 | PH_hs_0014938 | 0.0497 | 1.024 | 1.203 | 0.851 |
| THRAP6 | PH_hs_0016546 | 0.0333 | 1.144 | 1.346 | 0.850 |
| C14orf124 | PH_hs_0014609 | 0.081 | 0.927 | 1.091 | 0.849 |
| ZER1 | PH_hs_0002543 | 0.0669 | 1.173 | 1.384 | 0.848 |
| CENPC1 | PH_hs_0030005 | 0.0794 | 1.029 | 1.216 | 0.846 |
| MSL2L1 | PH_hs_0026964 | 0.0531 | 1.526 | 1.805 | 0.845 |
| RERE | PH_hs_0011475 | 0.091 | 1.759 | 2.082 | 0.845 |
| C11orf61 | PH_hs_0002997 | 0.0656 | 0.703 | 0.833 | 0.844 |
| CARD10 | PH_hs_0031262 | 0.0425 | 0.733 | 0.868 | 0.843 |
| MTMR2 | PH_hs_0010729 | 0.0105 | 0.890 | 1.056 | 0.843 |
| PAFAH2 | PH_hs_0026461 | 0.0656 | 0.957 | 1.135 | 0.843 |
| CCDC16 | PH_hs_0033231 | 0.0484 | 1.188 | 1.411 | 0.842 |
| C10orf4 | PH_hs_0023627 | 0.0762 | 0.876 | 1.041 | 0.842 |
| PA2G4 | PH_hs_0018140 | 0.1 | 0.637 | 0.757 | 0.842 |
| TPT1 | PH_hs_0024238 | 0.0087 | 1.769 | 2.100 | 0.842 |
| ZNF37A | PH_hs_0026090 | 0.0429 | 0.957 | 1.138 | 0.841 |
| KIAA0195 | PH_hs_0015513 | 0.098 | 0.717 | 0.852 | 0.841 |
| RMND5A | PH_hs_0027152 | 0.0389 | 0.909 | 1.083 | 0.840 |
| ST8SIA2 | PH_hs_0038039 | 0.0092 | 0.824 | 0.982 | 0.840 |
| RNF122 | PH_hs_0029232 | 0.0425 | 1.960 | 2.336 | 0.839 |
| NHP2L1 | PH_hs_0010764 | 0.0712 | 0.757 | 0.903 | 0.839 |
| PPIF | PH_hs_0018569 | 0.0588 | 0.719 | 0.858 | 0.838 |
| C21orf51 | PH_hs_0023831 | 0.0952 | 0.716 | 0.856 | 0.837 |
| ARF6 | PH_hs_0030844 | 0.0956 | 1.377 | 1.645 | 0.837 |
| USP20 | PH_hs_0000562 | 0.052 | 0.938 | 1.121 | 0.836 |
| YPEL1 | PH_hs_0027143 | 0.0652 | 1.126 | 1.347 | 0.836 |
| STXBP5L | PH_hs_0022365 | 0.028 | 0.956 | 1.144 | 0.836 |
| TRADD | PH_hs_0005024 | 0.0305 | 2.074 | 2.482 | 0.836 |
| MIS12 | PH_hs_0013705 | 0.0952 | 0.737 | 0.882 | 0.835 |
| KLHL21 | PH_hs_0000800 | 0.0833 | 1.068 | 1.281 | 0.834 |
| CBFA2T2 | PH_hs_0009258 | 0.0712 | 0.744 | 0.893 | 0.834 |
| AMACR,C1QTNF3 | PH_hs_0022141 | 0.0555 | 0.764 | 0.917 | 0.834 |
| POU2F1 | PH_hs_0014636 | 0.0633 | 0.640 | 0.768 | 0.833 |
| BDKRB1 | PH_hs_0003282 | 0.0864 | 0.389 | 0.468 | 0.832 |
| TCF20 | PH_hs_0011832 | 0.0613 | 1.115 | 1.341 | 0.832 |
| None | PH_hs_0019824 | 0.0273 | 1.120 | 1.347 | 0.832 |
| LETM2 | PH_hs_0023518 | 0.0305 | 0.786 | 0.946 | 0.831 |
| CHCHD7 | PH_hs_0025544 | 0.0902 | 1.307 | 1.574 | 0.831 |
| COG1 | PH_hs_0014607 | 0.0843 | 0.873 | 1.052 | 0.831 |
| PSCD3 | PH_hs_0000840 | 0.0529 | 0.382 | 0.460 | 0.830 |
| VHL | PH_hs_0033268 | 0.0253 | 1.215 | 1.467 | 0.828 |
| COX11 | PH_hs_0020619 | 0.0954 | 0.815 | 0.985 | 0.828 |
| C3orf62 | PH_hs_0031525 | 0.0695 | 0.937 | 1.135 | 0.826 |
| C12orf47 | PH_hs_0027012 | 0.052 | 1.104 | 1.338 | 0.825 |
| KISS1 | PH_hs_0005191 | 0.0712 | 0.974 | 1.181 | 0.825 |
| FAM102A | PH_hs_0032775 | 0.052 | 1.341 | 1.627 | 0.824 |
| GPD1L | PH_hs_0004724 | 0.0928 | 0.606 | 0.735 | 0.824 |
| FNBP4 | PH_hs_0000693 | 0.0389 | 0.693 | 0.841 | 0.824 |
| MRPL43 | PH_hs_0025152 | 0.0675 | 0.864 | 1.051 | 0.823 |
| MBIP | PH_hs_0024077 | 0.0389 | 0.582 | 0.708 | 0.822 |
| TMEM42 | PH_hs_0020042 | 0.0497 | 0.765 | 0.930 | 0.822 |
| SFRS5 | PH_hs_0010038 | 0.0635 | 1.348 | 1.640 | 0.822 |
| LPXN | PH_hs_0027124 | 0.027 | 2.307 | 2.807 | 0.822 |
| SMAD3 | PH_hs_0032513 | 0.0545 | 0.711 | 0.865 | 0.821 |
| RPL22 | PH_hs_0029744 | 0.0712 | 0.766 | 0.933 | 0.821 |
| C1orf9 | PH_hs_0006014 | 0.0296 | 0.663 | 0.809 | 0.819 |
| BTG2 | PH_hs_0004330 | 0.0866 | 2.928 | 3.573 | 0.819 |
| EXOC8 | PH_hs_0017264 | 0.0671 | 1.398 | 1.712 | 0.817 |
| PAQR8 | PH_hs_0012898 | 0.0897 | 1.113 | 1.364 | 0.816 |
| MARS2 | PH_hs_0006388 | 0.0954 | 0.462 | 0.567 | 0.814 |
| TARDBP | PH_hs_0015468 | 0.0711 | 0.681 | 0.837 | 0.813 |
| RPL4 | PH_hs_0033617 | 0.0897 | 0.641 | 0.790 | 0.811 |
| FOXO1A | PH_hs_0010156 | 0.0714 | 1.611 | 1.989 | 0.810 |
| ZIK1 | PH_hs_0027487 | 0.0954 | 0.800 | 0.988 | 0.810 |
| EIF4A2 | PH_hs_0030638 | 0.0943 | 0.804 | 0.993 | 0.810 |
| None | PH_hs_0035668 | 0.0954 | 0.745 | 0.920 | 0.809 |
| None | PH_hs_0017379 | 0.0389 | 1.801 | 2.228 | 0.809 |
| CERK | PH_hs_0002840 | 0.0618 | 1.269 | 1.570 | 0.808 |
| PDE3B | PH_hs_0016568 | 0.0505 | 1.220 | 1.510 | 0.808 |
| MAL | PH_hs_0004629 | 0.0963 | 0.887 | 1.098 | 0.807 |
| C7orf36 | PH_hs_0004756 | 0.0919 | 0.294 | 0.365 | 0.806 |
| ABCA8 | PH_hs_0022153 | 0.0873 | 1.029 | 1.279 | 0.804 |
| NPAT | PH_hs_0027183 | 0.0711 | 0.920 | 1.145 | 0.804 |
| None | PH_hs_0001875 | 0.0351 | 1.129 | 1.405 | 0.803 |
| HIVEP2 | PH_hs_0004266 | 0.0833 | 1.515 | 1.887 | 0.803 |
| B3GNT6 | PH_hs_0032812 | 0.0763 | 0.567 | 0.707 | 0.802 |
| ZNF227 | PH_hs_0024179 | 0.0226 | 0.933 | 1.163 | 0.801 |
| CCDC104 | PH_hs_0002032 | 0.0435 | 0.493 | 0.617 | 0.800 |
| TTF1 | PH_hs_0003596 | 0.0484 | 0.561 | 0.701 | 0.800 |
| HSPA8 | PH_hs_0040139 | 0.0541 | 0.769 | 0.963 | 0.799 |
| RPL13A | PH_hs_0033702 | 0.0495 | 1.037 | 1.300 | 0.798 |
| MFNG | PH_hs_0024190 | 0.0279 | 2.001 | 2.510 | 0.797 |
| SS18L2 | PH_hs_0030978 | 0.0227 | 1.107 | 1.391 | 0.796 |
| TAF1 | PH_hs_0000155 | 0.0892 | 0.816 | 1.025 | 0.796 |
| CDKN1B | PH_hs_0012886 | 0.0541 | 2.210 | 2.777 | 0.796 |
| FA2H | PH_hs_0022486 | 0.0389 | 0.749 | 0.941 | 0.795 |
| SYNCRIP | PH_hs_0039330 | 0.0919 | 0.517 | 0.650 | 0.795 |
| PPIH | PH_hs_0028751 | 0.0852 | 0.493 | 0.620 | 0.795 |
| None | PH_hs_0022237 | 0.0273 | 0.807 | 1.016 | 0.794 |
| THUMPD1 | PH_hs_0013727 | 0.0273 | 0.990 | 1.247 | 0.794 |
| PLD4 | PH_hs_0024691 | 0.0105 | 1.152 | 1.452 | 0.793 |
| THAP7 | PH_hs_0026853 | 0.0087 | 0.610 | 0.769 | 0.793 |
| BBS2 | PH_hs_0023845 | 0.0375 | 0.634 | 0.799 | 0.793 |
| TPT1 | PH_hs_0038568 | 0.0153 | 1.371 | 1.728 | 0.793 |
| SRPK2 | PH_hs_0007501 | 0.0954 | 1.814 | 2.289 | 0.793 |
| SFRS2B | PH_hs_0031886 | 0.0866 | 0.942 | 1.189 | 0.792 |
| C17orf48 | PH_hs_0028892 | 0.0712 | 1.365 | 1.724 | 0.792 |
| None | PH_hs_0019327 | 0.081 | 0.433 | 0.546 | 0.792 |
| CDC37L1 | PH_hs_0021997 | 0.0273 | 0.932 | 1.182 | 0.789 |
| NKTR | PH_hs_0024116 | 0.0956 | 0.824 | 1.045 | 0.788 |
| None | PH_hs_0024869 | 0.0226 | 1.755 | 2.227 | 0.788 |
| GDF15 | PH_hs_0027358 | 0.0429 | 0.619 | 0.786 | 0.788 |
| LPIN1 | PH_hs_0004665 | 0.0862 | 0.546 | 0.693 | 0.787 |
| CCDC16 | PH_hs_0024957 | 0.0111 | 1.733 | 2.203 | 0.787 |
| C12orf65 | PH_hs_0023662 | 0.0099 | 0.753 | 0.959 | 0.786 |
| RASSF1 | PH_hs_0002383 | 0.0405 | 1.345 | 1.712 | 0.785 |
| LIPH | PH_hs_0004088 | 0.0435 | 1.014 | 1.291 | 0.785 |
| RBM14,TMEM137 | PH_hs_0026880 | 0.0632 | 0.697 | 0.889 | 0.784 |
| RORC,LINGO4 | PH_hs_0034663 | 0.0712 | 0.809 | 1.032 | 0.783 |
| CHMP7 | PH_hs_0000516 | 0.0409 | 0.814 | 1.041 | 0.782 |
| C9orf123 | PH_hs_0000778 | 0.0497 | 0.905 | 1.157 | 0.782 |
| RPL22 | PH_hs_0032674 | 0.0862 | 0.674 | 0.863 | 0.780 |
| SLAMF6 | PH_hs_0015230 | 0.0712 | 2.869 | 3.676 | 0.780 |
| RALBP1 | PH_hs_0004329 | 0.0783 | 1.110 | 1.423 | 0.780 |
| LEPROTL1 | PH_hs_0022619 | 0.0273 | 1.023 | 1.311 | 0.780 |
| CYP2A13 | PH_hs_0032038 | 0.0305 | 0.812 | 1.042 | 0.779 |
| RPL18 | PH_hs_0035920 | 0.0614 | 0.801 | 1.030 | 0.778 |
| C1orf156 | PH_hs_0002813 | 0.0583 | 0.898 | 1.157 | 0.776 |
| PI16 | PH_hs_0002234 | 0.052 | 0.739 | 0.954 | 0.774 |
| C15orf29 | PH_hs_0022353 | 0.0794 | 1.830 | 2.367 | 0.773 |
| ZNF22 | PH_hs_0006015 | 0.0954 | 0.865 | 1.119 | 0.773 |
| CABC1 | PH_hs_0014100 | 0.0656 | 1.071 | 1.387 | 0.772 |
| MTR | PH_hs_0040560 | 0.0998 | 0.581 | 0.753 | 0.771 |
| HABP4 | PH_hs_0015569 | 0.0168 | 0.531 | 0.689 | 0.770 |
| TMEM66 | PH_hs_0028090 | 0.0947 | 2.451 | 3.186 | 0.769 |
| SORT1 | PH_hs_0002295 | 0.0545 | 1.321 | 1.718 | 0.769 |
| NEK7 | PH_hs_0040147 | 0.0954 | 1.900 | 2.471 | 0.769 |
| CREG2 | PH_hs_0022083 | 0.0799 | 1.113 | 1.450 | 0.768 |
| SNRPD3 | PH_hs_0024004 | 0.0712 | 1.014 | 1.321 | 0.768 |
| None | PH_hs_0001403 | 0.0541 | 0.289 | 0.377 | 0.767 |
| PITPNB | PH_hs_0011181 | 0.0425 | 0.727 | 0.947 | 0.767 |
| MRFAP1L1 | PH_hs_0030756 | 0.0022 | 1.359 | 1.772 | 0.767 |
| SPN | PH_hs_0026412 | 0.0429 | 2.000 | 2.608 | 0.767 |
| JARID2 | PH_hs_0003026 | 0.0351 | 1.219 | 1.592 | 0.766 |
| RPS23 | PH_hs_0029995 | 0.0816 | 0.621 | 0.811 | 0.766 |
| ARL4C | PH_hs_0029090 | 0.0265 | 1.590 | 2.076 | 0.766 |
| VPS13A | PH_hs_0003548 | 0.0168 | 0.656 | 0.857 | 0.766 |
| FAM76B | PH_hs_0014958 | 0.0952 | 1.552 | 2.029 | 0.765 |
| KIAA1128 | PH_hs_0027167 | 0.0095 | 1.250 | 1.634 | 0.765 |
| None | PH_hs_0032844 | 0.0327 | 0.739 | 0.966 | 0.764 |
| ZKSCAN1 | PH_hs_0022051 | 0.0541 | 0.762 | 0.997 | 0.764 |
| RABGAP1L | PH_hs_0012972 | 0.0467 | 2.302 | 3.019 | 0.763 |
| None | PH_hs_0000687 | 0.052 | 0.856 | 1.124 | 0.762 |
| CWF19L2 | PH_hs_0002146 | 0.0041 | 0.925 | 1.216 | 0.761 |
| MGAT5 | PH_hs_0038922 | 0.0429 | 0.813 | 1.070 | 0.760 |
| PPTC7 | PH_hs_0001326 | 0.052 | 2.695 | 3.551 | 0.759 |
| PPARBP | PH_hs_0001448 | 0.009 | 0.794 | 1.047 | 0.758 |
| DUSP16 | PH_hs_0001766 | 0.0211 | 0.888 | 1.172 | 0.758 |
| GOLGA4 | PH_hs_0020279 | 0.0711 | 0.581 | 0.768 | 0.758 |
| CCNI | PH_hs_0037641 | 0.0256 | 1.294 | 1.708 | 0.757 |
| RPS25 | PH_hs_0030699 | 0.0273 | 0.792 | 1.046 | 0.757 |
| DUSP8 | PH_hs_0026661 | 0.0405 | 0.496 | 0.656 | 0.756 |
| TBCC | PH_hs_0004267 | 0.0041 | 1.740 | 2.301 | 0.756 |
| TCF7 | PH_hs_0010094 | 0.0216 | 1.155 | 1.528 | 0.756 |
| RPS14 | PH_hs_0029971 | 0.0409 | 1.185 | 1.569 | 0.755 |
| TMCC1 | PH_hs_0017382 | 0.029 | 1.892 | 2.507 | 0.755 |
| RPL30 | PH_hs_0030028 | 0.0381 | 0.813 | 1.079 | 0.754 |
| TTLL1 | PH_hs_0025038 | 0.0105 | 0.759 | 1.007 | 0.754 |
| TMEM181 | PH_hs_0022239 | 0.0253 | 0.369 | 0.490 | 0.752 |
| ANKS3 | PH_hs_0017107 | 0.0253 | 0.471 | 0.627 | 0.752 |
| C1orf27 | PH_hs_0024332 | 0.0216 | 0.628 | 0.836 | 0.750 |
| ZNF331 | PH_hs_0008852 | 0.0354 | 0.561 | 0.749 | 0.749 |
| FAM129C | PH_hs_0020186 | 0.0554 | 1.022 | 1.369 | 0.747 |
| FANCF | PH_hs_0030800 | 0.0544 | 0.738 | 0.989 | 0.746 |
| RPS13 | PH_hs_0036044 | 0.0563 | 0.649 | 0.872 | 0.744 |
| RAPGEF6,FNIP1 | PH_hs_0025351 | 0.001 | 1.186 | 1.595 | 0.744 |
| ITGAE | PH_hs_0021633 | 0.0633 | 0.890 | 1.198 | 0.743 |
| ITM2A | PH_hs_0001556 | 0.0256 | 0.640 | 0.863 | 0.742 |
| FYN | PH_hs_0024531 | 0.0833 | 2.085 | 2.816 | 0.740 |
| MAGEH1 | PH_hs_0014406 | 0.0308 | 0.876 | 1.185 | 0.739 |
| SGK | PH_hs_0015329 | 0.0714 | 0.537 | 0.727 | 0.739 |
| RHOH | PH_hs_0006061 | 0.0979 | 1.621 | 2.194 | 0.739 |
| C17orf54 | PH_hs_0026419 | 0.0613 | 1.179 | 1.599 | 0.737 |
| VSIG9 | PH_hs_0025150 | 0.0296 | 2.259 | 3.065 | 0.737 |
| PIK3R1 | PH_hs_0021977 | 0.0531 | 1.624 | 2.206 | 0.736 |
| BIN1 | PH_hs_0011389 | 0.0339 | 1.423 | 1.935 | 0.735 |
| STAT4 | PH_hs_0004637 | 0.0928 | 2.372 | 3.229 | 0.735 |
| SLC25A38 | PH_hs_0000846 | 0.0354 | 0.579 | 0.788 | 0.734 |
| KIAA1509 | PH_hs_0022063 | 0.0405 | 1.670 | 2.280 | 0.732 |
| None | PH_hs_0032374 | 0.0783 | 0.638 | 0.871 | 0.732 |
| TXNL5 | PH_hs_0038183 | 0.0897 | 1.193 | 1.630 | 0.732 |
| CD2 | PH_hs_0004986 | 0.0712 | 4.295 | 5.877 | 0.731 |
| PTGES3 | PH_hs_0040316 | 0.0253 | 0.458 | 0.627 | 0.731 |
| None | PH_hs_0006606 | 0.0041 | 1.885 | 2.580 | 0.731 |
| GAL3ST4,GPC2 | PH_hs_0003251 | 0.0345 | 0.584 | 0.801 | 0.730 |
| C12orf57 | PH_hs_0019220 | 0.0253 | 0.477 | 0.654 | 0.729 |
| RNF125 | PH_hs_0031817 | 0.0927 | 1.510 | 2.074 | 0.728 |
| INPP4B | PH_hs_0009243 | 0.0614 | 0.653 | 0.899 | 0.726 |
| None | PH_hs_0029467 | 0.0256 | 0.608 | 0.838 | 0.725 |
| RNF157 | PH_hs_0025329 | 0.0253 | 0.644 | 0.890 | 0.724 |
| GOLGA8A,GOLGA8B | PH_hs_0028226 | 0.0256 | 0.703 | 0.972 | 0.724 |
| OCIAD2 | PH_hs_0005218 | 0.0216 | 0.728 | 1.005 | 0.724 |
| CCDC66 | PH_hs_0023634 | 0.0253 | 0.898 | 1.245 | 0.721 |
| PBXIP1 | PH_hs_0026572 | 0.0093 | 2.520 | 3.494 | 0.721 |
| MCRS1 | PH_hs_0039797 | 0.0954 | 0.722 | 1.002 | 0.721 |
| None | PH_hs_0012483 | 0.0041 | 0.520 | 0.724 | 0.718 |
| RABGAP1L | PH_hs_0040406 | 0.035 | 2.111 | 2.939 | 0.718 |
| RPLP2 | PH_hs_0030250 | 0.052 | 0.839 | 1.169 | 0.718 |
| CREB1 | PH_hs_0022033 | 0.0712 | 2.166 | 3.029 | 0.715 |
| Ube2b-rs1 | PH_hs_0022211 | 0.0041 | 0.303 | 0.424 | 0.715 |
| ASF1A | PH_hs_0023538 | 0.0712 | 0.944 | 1.320 | 0.715 |
| C6orf190 | PH_hs_0018474 | 0.0253 | 0.769 | 1.077 | 0.714 |
| TRAF3IP3 | PH_hs_0022629 | 0.0588 | 6.162 | 8.637 | 0.713 |
| 6-Sep | PH_hs_0005067 | 0.035 | 0.828 | 1.164 | 0.712 |
| ZNF91 | PH_hs_0030970 | 0.0541 | 0.903 | 1.276 | 0.708 |
| LILRA4 | PH_hs_0024682 | 0.0614 | 0.803 | 1.135 | 0.707 |
| IL11RA,GALT | PH_hs_0023030 | 0.0305 | 1.984 | 2.808 | 0.706 |
| TIGD3 | PH_hs_0003426 | 0.0358 | 1.920 | 2.721 | 0.706 |
| KLRC4,KLRK1 | PH_hs_0033086 | 0.0253 | 1.602 | 2.276 | 0.704 |
| PDCD4 | PH_hs_0032355 | 0.0256 | 1.825 | 2.595 | 0.703 |
| BSG | PH_hs_0026427 | 0.0647 | 1.651 | 2.350 | 0.702 |
| CR2 | PH_hs_0031301 | 0.0471 | 0.800 | 1.139 | 0.702 |
| DCK | PH_hs_0027522 | 0.0865 | 0.983 | 1.402 | 0.701 |
| ITGA4 | PH_hs_0022595 | 0.0695 | 2.082 | 2.979 | 0.699 |
| TRAF5 | PH_hs_0024370 | 0.0253 | 0.908 | 1.300 | 0.698 |
| THEM4 | PH_hs_0032546 | 0.0022 | 0.762 | 1.094 | 0.696 |
| PIK3C2A | PH_hs_0013220 | 0.0763 | 0.545 | 0.786 | 0.693 |
| TGFBR3 | PH_hs_0016619 | 0.0712 | 0.561 | 0.811 | 0.692 |
| BANK1 | PH_hs_0011427 | 0.0105 | 1.885 | 2.726 | 0.691 |
| CD7 | PH_hs_0002921 | 0.0273 | 0.962 | 1.391 | 0.691 |
| C13orf15 | PH_hs_0004423 | 0.0792 | 2.994 | 4.334 | 0.691 |
| SSBP2 | PH_hs_0039403 | 0.0253 | 0.684 | 0.992 | 0.689 |
| KLRG1 | PH_hs_0008729 | 0.0656 | 1.915 | 2.784 | 0.688 |
| ANK3 | PH_hs_0025787 | 0.0041 | 0.396 | 0.578 | 0.686 |
| RPS6KA5 | PH_hs_0006029 | 0.0216 | 2.190 | 3.215 | 0.681 |
| C9orf48 | PH_hs_0030423 | 0.0866 | 0.618 | 0.912 | 0.677 |
| None | PH_hs_0012342 | 0.0712 | 0.591 | 0.873 | 0.677 |
| CYLD | PH_hs_0024407 | 0.0187 | 3.192 | 4.717 | 0.677 |
| ALDH1A1 | PH_hs_0004409 | 0.0447 | 0.695 | 1.031 | 0.674 |
| LBH | PH_hs_0003682 | 0.001 | 1.657 | 2.470 | 0.671 |
| NFKBIB | PH_hs_0030976 | 0.0852 | 3.022 | 4.506 | 0.671 |
| SPOCK2 | PH_hs_0020492 | 0.0355 | 4.661 | 6.964 | 0.669 |
| CCR5 | PH_hs_0031237 | 0.0216 | 2.830 | 4.264 | 0.664 |
| MTAC2D1 | PH_hs_0003705 | 0.0588 | 1.664 | 2.510 | 0.663 |
| UBASH3A | PH_hs_0010839 | 0.052 | 2.064 | 3.119 | 0.662 |
| AKT3 | PH_hs_0023601 | 0.0139 | 0.478 | 0.723 | 0.660 |
| PRF1 | PH_hs_0000291 | 0.0979 | 6.344 | 9.730 | 0.652 |
| PKIA | PH_hs_0020144 | 0.0141 | 0.725 | 1.116 | 0.650 |
| PLEKHA1 | PH_hs_0014922 | 0.0586 | 0.498 | 0.767 | 0.649 |
| AQP3 | PH_hs_0012796 | 0.0036 | 0.719 | 1.115 | 0.645 |
| CD1C | PH_hs_0000177 | 0.0273 | 2.568 | 3.984 | 0.645 |
| LRRC8C | PH_hs_0002135 | 0.0205 | 0.661 | 1.026 | 0.644 |
| GZMA | PH_hs_0005055 | 0.0273 | 10.607 | 16.653 | 0.637 |
| SH2D1A | PH_hs_0009133 | 0.0083 | 1.351 | 2.122 | 0.636 |
| PTPN4 | PH_hs_0023771 | 0.0105 | 1.971 | 3.119 | 0.632 |
| CD5 | PH_hs_0003778 | 0.028 | 2.720 | 4.309 | 0.631 |
| PTPRCAP,CORO1B | PH_hs_0009399 | 0.0712 | 2.180 | 3.455 | 0.631 |
| None | PH_hs_0002354 | 0.0095 | 3.716 | 5.908 | 0.629 |
| LCK | PH_hs_0000240 | 0.0087 | 1.855 | 2.950 | 0.629 |
| LY9 | PH_hs_0024619 | 0.0151 | 3.269 | 5.198 | 0.629 |
| CD3G | PH_hs_0000306 | 0.0956 | 2.226 | 3.563 | 0.625 |
| LEF1 | PH_hs_0003252 | 0.0273 | 0.661 | 1.060 | 0.624 |
| CA5B | PH_hs_0039389 | 0.0095 | 0.822 | 1.318 | 0.624 |
| CD3D | PH_hs_0005206 | 0.0201 | 1.277 | 2.068 | 0.618 |
| TRAT1 | PH_hs_0031781 | 0.0693 | 4.716 | 7.688 | 0.614 |
| LAT | PH_hs_0026523 | 0.0187 | 2.093 | 3.432 | 0.610 |
| KIAA0748 | PH_hs_0002788 | 0.0854 | 0.994 | 1.644 | 0.605 |
| CD3G | PH_hs_0030882 | 0.0041 | 2.150 | 3.561 | 0.604 |
| GPR115 | PH_hs_0022672 | 0.0588 | 0.710 | 1.191 | 0.596 |
| VSIG9 | PH_hs_0033271 | 0.0168 | 2.406 | 4.081 | 0.589 |
| TRAV20,TRDV2 | PH_hs_0036583 | 0.0675 | 7.238 | 12.471 | 0.580 |
| CD160 | PH_hs_0004672 | 0.0049 | 1.234 | 2.131 | 0.579 |
| DYRK2 | PH_hs_0010380 | 0.0041 | 1.385 | 2.416 | 0.573 |
| EBI2 | PH_hs_0040414 | 0.0425 | 4.707 | 8.266 | 0.569 |
| MS4A1 | PH_hs_0025653 | 0.0151 | 2.022 | 3.608 | 0.560 |
| CCR3 | PH_hs_0026576 | 0.0614 | 6.766 | 12.251 | 0.552 |
| EDG1 | PH_hs_0009283 | 0.0256 | 2.561 | 4.730 | 0.542 |
| FCER1A | PH_hs_0000108 | 0.0584 | 14.549 | 27.554 | 0.528 |
| EBI2 | PH_hs_0000092 | 0.001 | 3.447 | 6.651 | 0.518 |
| GRAMD1C | PH_hs_0037695 | 0.0087 | 1.227 | 2.370 | 0.518 |
